# Oligosaccharides: a boon from nature’s desk

**DOI:** 10.1186/s13568-016-0253-5

**Published:** 2016-10-03

**Authors:** Seema A. Belorkar, A. K. Gupta

**Affiliations:** 1 Department of Microbiology and Bioinformatics, Bilaspur University, 206, Budhiya complex, Sarkanda, Bilaspur, Chhattisgarh 495004 India; 2 Pt. Ravishankar Shukla University, Raipur, CG 492010 India

**Keywords:** Oligosaccharides, Prebiotics, Functional food and applications

## Abstract

This article reviews the varied sources of oligosaccharides available in nature as silent health promoting, integral ingredients of plants as well as animal products like honey and milk. The article focuses on exotic and unfamiliar oligosaccharides like Galactooligosaccharides, Lactulose derived Galactooligosaccharides, Xylooligosaccharides, Arabinooligosaccharides and algae derived Marine oligosaccharides along with the most acknowledged prebiotic fructooligosaccharides. The oligosaccharides are named as on the grounds of the monomeric units forming oligomers with functional properties. The chemical structures, natural sources, microbial enzyme mediated synthesis and physiological effects are discussed. An elaborate account of the different types of oligosaccharides with special reference to fructooligosaccharides are presented. Finally, the profound health benefits of oligosaccharides are rigourously discussed limelighting its positive physiological sequel.

## Introduction

Food industry is presently witnessing an upcoming market for edible products having health benefits apart from nutrition, now well recognized as functional foods. The market of functional foods is facing an increasing demand also because of consumer awareness about health. According to the Global Industry Analyst (GIA) report on the demand of prebiotics, based on studies in market trends in countries like US, Canada, Japan, Europe (France, Germany, Italy, UK, Spain, Russia and rest of Europe), Asia–Pacific (China, India and Rest of Asia–Pacific) and rest of World, the industry is likely to flourish to a tune of US $4.8 billion by 2018 from US $1.0 billion in 2011 (Spinner 2013).

Japan is one of the leading countries giving importance to functional food market focusing on “Food of Specified Health Use” (FOSHU). Many European countries like Germany, France, United Kingdom and Netherlands have also showed an extended demand for functional foods (Katapodis et al. 2004; Menrad 2003).

Since past three decades there has been constant evaluation of market trend of western countries witnessing increased demand of functional foods. Even in developing country like India, where the dairy industry is one of the main industries supporting economy, there has been a significant rise in demand of value added dairy products encompassing health benefits to the consumers (Gour 2013).

Prebiotics and probiotics have raised as best option for quench of the increasing need of functional food. Roberfroid (2000) studied probiotics and prebiotics food and reviewed their properties to be rightly labeled as functional foods. He explained that prebiotics are non-digestible food ingredients that benefit the host by selectively stimulating the growth or activity of one or limited number of bacteria in colon.

Food ingredients which naturally offer resistance to digestion, when reach the intestine exhibit a favoring effect on normal flora of the colon are called as prebiotics. Prebiotics encompass several health benefits like the calorie-free nature, act as artificial sweeteners, have non-carcinogenic nature and stimulate the growth of *Bifidobacterium* and probiotic *Lactobacilli* in the colon (Saminathan et al. 2011). They possess preventive effect against colon cancer (Moore et al. 2003). They have ability to decrease cholesterol levels in the serum (Fernandez et al. 2003). Phospholipids and triglyceride levels are also found to be regulated in the serum by prebiotic food (Katapodis et al. 2004). Fructooligosaccharides (FOS) are gaining wide acceptance as prebiotics (Belorkar et al. 2013). This mini review presents an overview of the types of oligosaccharides existing in nature, their sources and major thrust applications.

## Oligosaccharides: types, sources and applications

**Fig. 1:**
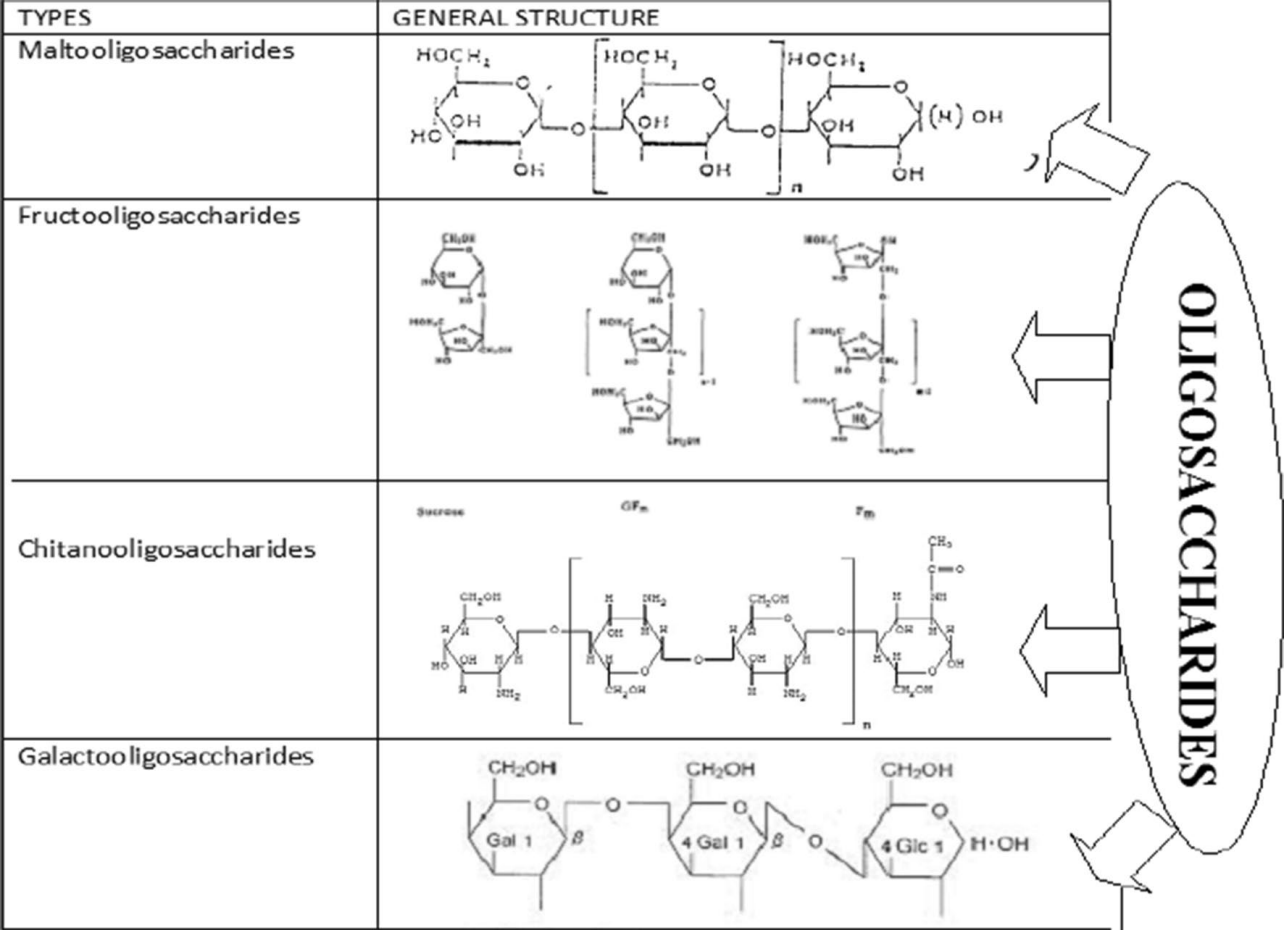
Overview of structure of some common oligosaccharides

Extensive research has been done on various types of oligosaccharides. They differ in their nature of monomeric sugars and are named so. They have varied sources of origin and differ in their benefits imparted to the consumer. The most popular oligosaccharides are FOS, Galactooligosaccharides (GOS), Lactulose derived galactooligosaccharides (LDGOS), Xylooligosaccharides (XOS), Arabinooligosaccharides (AOS), algae derived marine oligosaccharides (ADMO). Other oligosaccharides occurring in nature are Pectin-derived acidic oligosaccharides (pAOS), Maltooligosaccharides (MOS), Cyclodextrins (CD) and human milk oligosaccharides (HMO) with specific acknowledged benefits. The oligosaccharides have great industrial applications (Crittenden and Playne 1996; Prapulla et al. 2000). The chemical structure of some important oligosaccharides are given in Fig. 1.

## Structure of fructooligosaccharides

FOS consist of a fructose units polymerized to different extent. Oligomers with two fructose units are called as 1-kestose. Oligomers with three fructose units are called as 1-nystose. Oligomers with four fructose units are called as 1-fructofuranosyl-nystose. The sugars are linked by β-2, 1 position of sucrose (Sangeetha et al. 2005).

## Occurrence of FOS

Varieties of sources cater fructooligosaccharides in varying concentrations as its natural component like wheat, honey, onion, garlic and banana (Roberfroid and Slavin 2000). Barley and tomato contains 0.15 % of fructooligosaccharides. Banana and brown sugar has 0.30 % fructooligosaccharides. Honey has 0.75 % of fructooligosaccharides (Flamm et al. 2001).

Bornet et al. (2002) recorded the occurrence of short chain FOS in many edible plants. Fructooligosaccharides expresses degree of polymerization from 1 to 5 units of fructose. Short chain oligosaccharides are similar to dietary fibers in resisting digestion in intestine and getting converted to acetate, propionate, butyrate and gas in colon. Fructooligosaccharides also add up to the fecal matter and gives improved bowel movement. In the digestive tract they promote *Bifidobacterium* and on other hand have an inhibitory effect on *Clostridium perfringes* in colon.

**Fig. 2:**
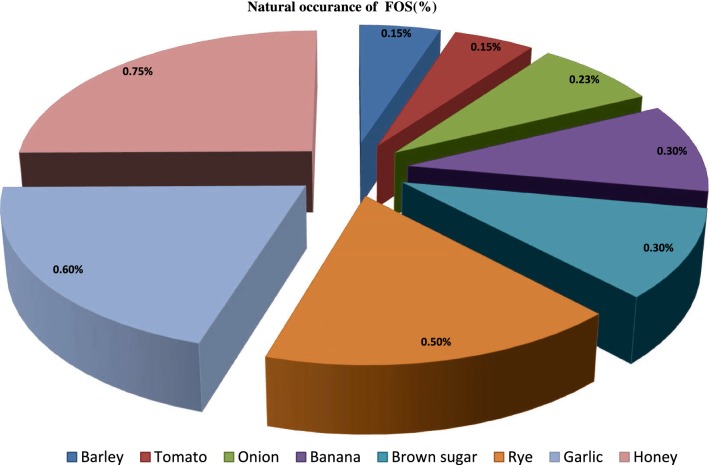
Distribution of FOS in various natural products

FOS are found abundantly in nature as a component of cereals, fruits and vegetables next to starch specified in Fig. 2 (Sangeetha et al. 2005). These exhibit resistance to basic enzymes involved in digestion like alpha amylase, saccharase and maltase when investigated in humans (Losada and Olleros 2002).

Johnson et al. (2013) reported that lentils are rich in prebiotics. There is a significant variation in prebiotic carbohydrate composition of different types of lentils. They analyzed Raffinose-family oligosaccharides, sugar alcohols, fructooligo-saccharide and resistant starch carbohydrates. They recorded the occurrence of Raffinose-family oligosaccharides, sugar alcohols, fructooligosaccharides and resistant starch as 4071, 1423, 62 and 7500 mg per 100 g dry matter, respectively.

## Fructosyltransferase enzyme

Some plants and microorganisms express fructosyltransferase enzyme naturally. The activity of this enzyme empowers these organisms to synthesize fructooligosaccharides (Sanchez et al. 2008). Fructosyltransferase enzyme from different sources exhibit different mechanisms of action and produce different mixtures of oligosaccharides.

## Beneficial health effects of FOS on consumers

FOS are receiving attention and importance not merely because of their application as alternative sweeteners but rather for the accompanied desirable characteristics. The earlier known health benefits of FOS were inhibitory effect on pathogens and stimulatory effect on *Bifidobacterium*. The FOS was analyzed further to highlight its detailed interaction with *Bifidobacterium* (probiotics) which paved a pathway for the concept of synbiotics (Perrin et al. 2001; Vander et al. 2004). The health benefits of FOS have been reviewed by many workers (Antosova and Polakovic 2001; Hernandez et al. 2009; Patel and Goyal 2011; Ganaje et al. 2014).

Some of the evident health benefits observed by consumption of fructooligosaccharides include the following:

### Promotes growth of the gut micro flora

Studies on *Bifidobacterium* species revealed that fructooligosaccharides preferred those carbohydrates which allow maximum growth and metabolic activities of this beneficial flora in human intestine (Palframan et al. 2003). The diet and its composition have an impact on gut and its microflora. It has been observed that any kind of change in the diet affects the metabolism of the inhabitants. The dietary fibres like oligosaccharides exert a combined effect on both the pH environment of the gut and the metabolism of bacterial community (Chen et al. 2000; Flint et al. 2007).

### Prebiotics have multidimensional effect on host-bacteria interaction

It is now well established fact that host bacteria interactions are highly specific with varied dimensions. The digestion resistant carbohydrates in the gut are fermented in the colon which causes increase in the serum lactate levels. The study was conducted on horses by injecting fructooligosaccharides directly in caecum and acidotic state was maintained. Its effect on caecum bacteria and metabolites were analyzed. *Streptococcal* species (EHSS) showed positive relation with caecum lactate and negative response with serum lactate; however, serum lactate has a positive influence on *Enterobacteriaceae* (Rudi 2010).

### Genetic features direct the probiotic effect of bacteria

Excellent studies on genomics of lactic acid bacteria in relation to their role in functional foods have been done by Klaenhammer et al. (2005). Their findings discovered that many genetic features exert control over the bacterial metabolic and probiotic process.

### Development of resistance to ill effects of bile salts

Fructooligosaccharides and their monomeric derivatives offer resistance against the ill effects of bile salts on Bifidus group of intestinal inhabitants. Perrin et al. (2001) studied the inhibitory effect of bile salts on three strains of *Bifidobacterium* in medium containing any carbohydrate source. In presence of fructooligosaccharides in the medium the *Bifidobacterium* improved their resistance and demonstrated better growth in presence of bile salts. Macfarlane et al. (2008) studied the effect of inulo, galacto and fructooligosaccharides was extremely favorable for *Bifidobacterium* and also *Lactobacilli* but to a lesser extent. Their health benefits encompass features like putative anti-cancer properties, mineral absorption, lipid metabolism, anti-inflammatory and other immune effects such as atopic disease.

### Promotes preferential growth of Bifidus

A statistical model was used by Shuhaimi et al. (2009) for the study of growth of *Bifidobacterium pseudocatenulatum* G4 under the influence of prebiotic. The physiological effect of inulin and fructooligosaccharides were investigated with sorbitol, arabinan and inoculum rate. Fractional factorial design was used to determine their effect on growth of selected bacterium in skimmed milk. They optimized their growth conditions and concluded that in 1 L fermentor, the yield increase and Central Composite Design was very effective in optimization of medium for growth of Bifidus. In a similar study, Ketabi and Dieleman (2011) investigated the effect of isomalto-oligosaccharides on intestinal microflora of rats and inferred that it specifically stimulated the growth of *Lactobacilli*.

### Removal of cholesterol

Cholesterol was found to be evidently removed by *Lactobacillus acidophilus* ATCC 4962 in the presence of prebiotics in a study conducted by Liong and Shah (2005). The effect of six prebiotics including fructooligosaccharides was used to investigate the best combination for effective removal of cholesterol. The first-order model, the second-order polynomial regression model and quadratic models were used in their study.

### Artificial sweetness

Apart from all the above stated prime health benefits fructooligosaccharides also has artificial sweetness and low caloric value. Artificial sweeteners are constantly in demand due to need of diabetics and health conscious consumers. Initially the demand was satisfied by aspartame agent or natural sweeteners like palatinose. Due to their popular use all types of oligosaccharides remained poorly exploited despite their functional properties (Mussatto et al. 2009).

### Role in osteoporosis

The most recent trial of fructooligosaccharides supplemented with calcium in post menopausal women have registered beneficial effects in bone mineral density which is highly significant in osteoporosis (Slevin et al. 2014).

## Galactooligosaccharides (GOS) and Lactulose derived galactooligosaccharides (LDGOS)

Mammalian milk is the natural source of GOS. Industrially trans galactosylation of lactose present in whey catalysed by β-galactosidases is gaining momentum as an promising alternative for synthesis of GOS (Affertsholt-Allen 2009).

β-Galactosidase is a hydrolase that attacks the o-glucosyl group of lactose. The general mechanism of enzymatic lactose hydrolysis has a transgalactosylic nature, involving a multitude of sequential reactions with disaccharides (other than lactose) and higher saccharides, collectively named galacto-oligosaccharides (GOS), as intermediate products (Wallenfels and Malhotra 1960; Goulas et al. 2007). Non digestible oligosaccharides have wider applications (Sako et al. 1999).

The GOS are complex mixtures of oligosaccharides ranging from two to eight moieties, and different glycosidic linkages: β-(1,1), β-(1,2), β-(1,3), β-(1,4) and β-(1,6) (Playne and Crittenden 2009). The hydrolytic enzymes preferentially expressed by *Bifidobacterium* species specifically target β-glycosidic linkages of GOS in the intestine (Macfarlane et al. 2008).

Microbes are exuberant sources of the enzymes producing Lactulose and GOS (Nguyen et al. 2009; Splechtna et al. 2006, 2007; Maischberger et al. 2008; Placier et al. 2009). The operation conditions are to be properly monitored for optimal ratio of lactulose and GOS for potential synthesis of prebiotics (Guerrero et al. 2013; 2015).

**Fig. 3:**
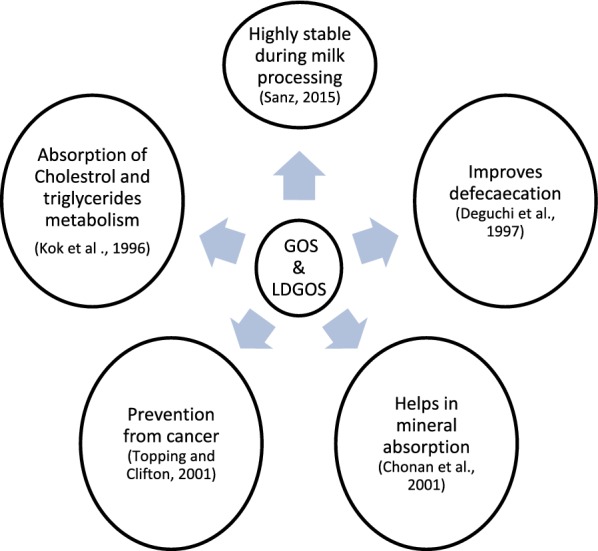
Functions of GOS and LDGOS

The main physiological effects of GOS are related with their composition and activities of the intestinal microbiota (Algieri et al. 2014). The human intestinal tract harbors a complex community of bacteria, eukaryotic microorganisms, archaea, viruses, and bacteriophages, collectively referred to as the intestinal microbiota. Bacteria account for the majority of these microorganisms: their total number in the human gut is estimated at 1014 cells mainly present in the colon (Backhed et al. 2005; Lupp and Finlay 2005). The wide applications of GOS and LDGOS are represented in Fig. 3.

## Xylooligosaccharides (XOS)

Xylooligosaccharides or feruloyl oligosaccharides are known to be produced by *Aspergillus*, *Trichoderma*, *Penicillium*, *Bacillus* and *Streptomyces*. It is found in plant sources like Bengalgram husk, wheat bran and straw, spentwood, barley hulls, brewery spent grains, almond shells, bamboo and corn cob. XOS mainly exerts prebiotic effect in consumers.

These unusual oligosaccharides are composed by chains of xylose moieties linked by β-(1,4) bonds, with a polymerization degree ranging from two to ten monosaccharides.

**Fig. 4:**
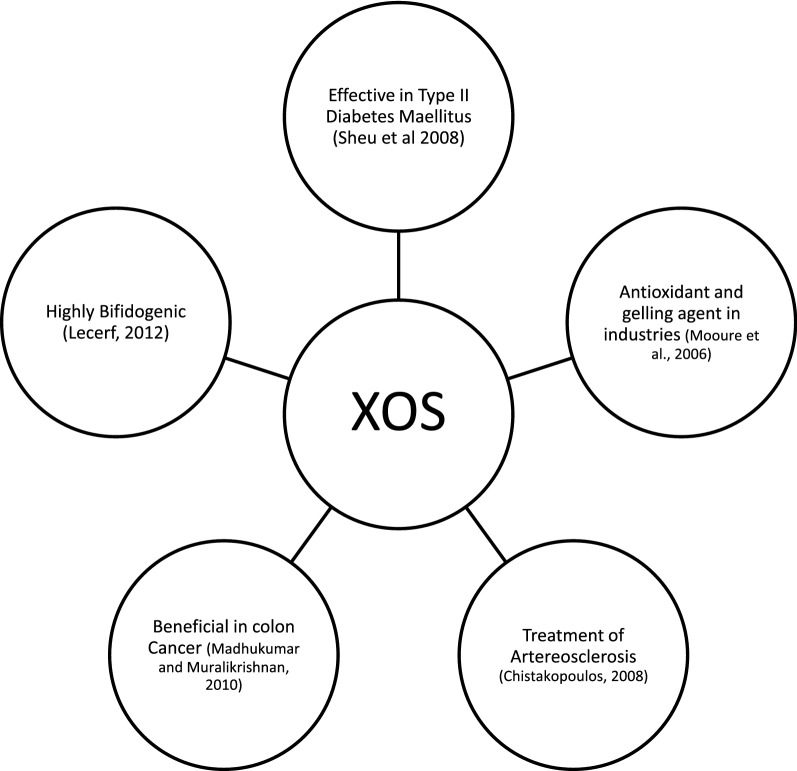
Functions of XOS

It is also known to act as a plant growth regulator. It has multidimensional applications as antioxidant and gelling agent in food products, beneficial for diabetes, in treatment of arteriosclerosis, reduces total cholesterol and LDL in patients with type 2 diabetes mellitus and in colon cancer (Chung et al. 2007; Sheu et al. 2008; Lecerf et al. 2012; Moure et al. 2006; Katapodis and Chistakopoulos 2008; Madhukumar and Muralikrishna 2010). Figure 4 is a diagrammatic representation of applications of XOS.

## Arabinooligosaccharides (AOS)

Arabinooligosaccharides are yet another class of oligosaccharides which hold the potential of being labelled as prebiotics. The exuberant source of AOS is arabinan polysaccharide a branched pectic polysaccaharide exhibiting linkage of 1,3 and 1,5 α l -arabinofuranosyl residues (Vogel 1991). Arabinose occurs naturally in arabinans, arabinogalactans or arabino xylans present in plants cell wall components. The nature of linkages differ depending upon the sources. The brush border epithelial cells of the intestine are inefficient to degrade the polysaccharides present in plant cell wall. This resistance of cell wall polysaccharides towards intestinal hydrolysis confer them the potential to be used as prebiotics (Yoo et al. 2012; Rastall and Hotchkiss 2003). The efficacy of the prebiotic effect of AOS is structure dependent (Casci et al. 2006; Gullón et al. 2011).

Initially the extraction was practiced by hot alkali treatment (Cibe 2003) of sugar beet dried pulp (5.5 million tons) a major coproduct of beet sugar industries residue in European countries.

AOS can also obtained by enzymatic hydrolysis of Arabinose containing polymers. Beldman et al. (1997) classified the Arabinan degrading enzymes in six classes-

(i) α-l-Arabinofuranosidase (EC 3.2.1.55), which is not active with polymers (Komae et al. 1982; Weinstein and Alber sheim 1979).
(ii) α-l-Arabinofuranosidase, which is active with polymers (Kaji and Tagawa, 1970; Rombouts et al. 1988).
(iii) α-l-Arabinofuranohydrolase, which is specific for arabinoxylans (Kormelink et al. 1991; Van Laere et al. 1997).
(iv) exo-α-l-Arabinanase, which is not active with *p*-nitrophenyl-α-l-arabinofuranoside (Kaji and Shimokawa^1984^; McKie et al. 1997).
(v) β-l-Arabinopyranosidase (Dey 1983; Kaji and Saheki 1975).
(vi) endo-1, 5-α-l-Arabinanase (EC 3.2.1.99) (Voragen et al. 1987).

The various degree of polymerization (dp) are obtained when subjected to ultrafiltration can produce Oligosaccharides of uniform molecular weight (Matsubara et al. 1996; Jian et al. 2013). AOS derived from sugar beet pectin (Al-Tamimi et al. 2006) and lemon peel (Hotchkiss et al. 2010) support the intestinal bifidus flora nearly equal to FOS and Inulooligosaccharides (Gómez et al. 2015; Palframan et al. 2002; Rycroft et al. 2001; Sanz et al. 2005). The extent of response is directly proportional to the dp of the oligosaccharide (Sulek et al. 2014; Westphal et al. 2010).

Apart from the normal benefits, AOS is reported to reduce the inflammatory conditions in Ulcerative colitis patients. Invitro experiments have proved about specific stimulation of *Bifidobacterium* and *Lactobacillus* accompanied by production of SCFA acetate which is well known stimulator of anti inflammatory response. AOS can prove to be a boon for patients suffering from Ulcerative colitis after in vivo confirmation (Vigsnæs et al. 2011).

Algal-oligosaccharides lysate (AOL) and neoagarooligosaccharides (NAOS) occur in the algal polysaccharide extracts (APEs) of *Gracilaria* and *Monostroma*and inenzymatic hydrolysis of agarose. They have a prebiotic effect and also act as an antioxidant (Wu et al. 2005; Hu et al. 2006).

## Algae-derived marine oligosaccharides

Recently, algae are reported to contain bipolysaccharides (Stengel et al. 2011; Barra et al. 2014). The bioactive components mainly include glucose, starch and other polysaccharides (Hamed et al. 2015). Besides these, oligosaccharides are another group of carbohydrates with small dp containing 3–10 sugar units, ranging from disaccharides and/or carbohydrates with up to 20 residues with defined functions (Patel and Goyal 2010).

The chemical structure and conformation decides the classification of algae-derived marine oligosaccharides namely chitosan-, laminarin-, alginate-, fucoidan-, carrageenan- and ulvan-oligosaccharides.

**Fig. 5:**
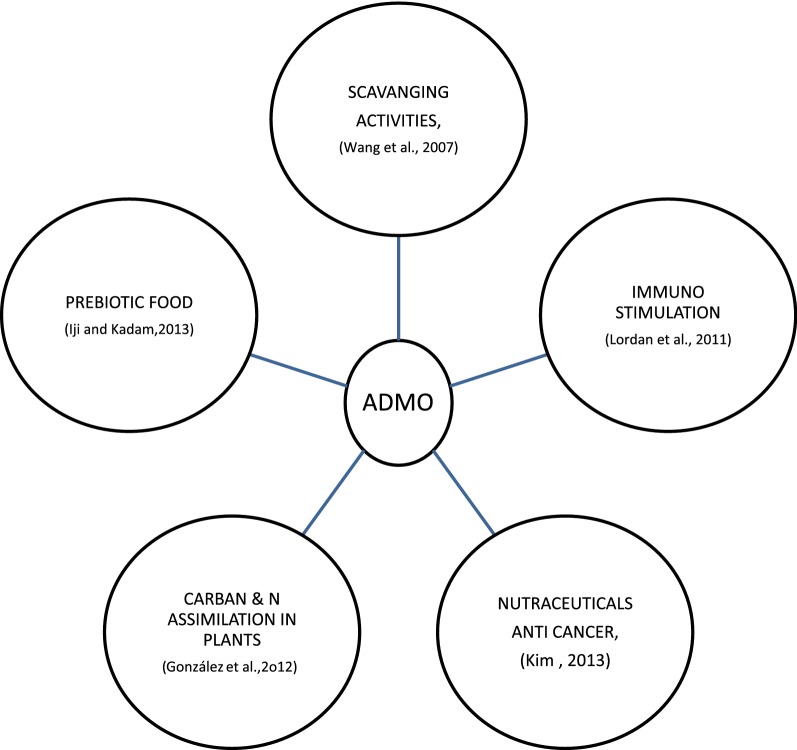
Functions of AOS

The note worthy bioactive compounds in Marine macroalgae or seaweeds is namely polysaccharides, tannins, and diterpenes. (O’sullivan et al. 2010). These ingredients may lead a pivitol role in nutraceuticals (Milinki et al. 2011). The functions of ADMO are given in Fig. 5.

## Other oligosaccharides

Mannanoligosaccharides (MOS) are mainly isolated from cell wall fragments of yeast. It was found to alter the gut microflora in fishes. It has been used as an alternative to antibiotics and added to improve the nutritive value of broiler diets (Dimitroglou et al. 2010; Eseceli et al. 2010). Chitosanoligosaccharides (COS) has been recorded to be produced by depolymerisation of chitosan. They are mainly used as an antioxidative agent, anti-tumor agent and anti-microbial agent. Chitosan oligosaccharides have been recorded to protect normal cells from apoptosis (Liu et al. 2010). Human milkoligosaccharide (HMO) naturally occurs in human breast milk. It signifies the preferential growth of *Bifidobacterium* and *Lactobacilli* in the colon of mother fed babies (Quigley 2010). Gentiooligosaccharides (GeOS) is produced by digestion of starch and mainly used as a prebiotic (Cote 2009; Fujimoto et al. 2009).

Pectin-derived acidic oligosaccharides (pAOS) occur in higher plant products like fruits and vegetables. It mainly finds its applications in infant formulae to subside diarrhoea, increase absorption of minerals and calcium ions and also has antioxidant effects (Liu et al. 2010). pAOS also successfully helped in the lung infections by modulating the intestinal microbiota and the inflammatory and immune responses (Bernard et al. 2015). Cyclodextrins (CDs) are produced by transformation of starch by *Bacillus macerans*. It is used as a stabilizer for volatile compounds in food preparations and chemicals. It acts as an antioxidant. It is used as taste enhancers in bitter medicines and food items (Astray et al. 2009; Courtois 2009).

Although all oligosaccharides are exhibiting prebiotic properties but fructo-oligosaccharides has gained much attention as artificial sweeteners because they provide sweet taste to the consumer and do not increase the blood glucose level. Therefore, they find important place in the food of diabetics. Thus, fructooligosaccharides act as artificial sweeteners with functional properties apart from sweetness similar to that of natural sweeteners.

Oligosaccharides from various sources have been considered as boon due to health benefits they encompass along with property of being used as an artificial sweetner. Due to the diversified health benefits conferred by them, they have earned a prominent recognition as Nutraceuticals presently limelighted in the health market. The microbial production of enzymes catering the catalysis of oligosaccharides are now targeted by the biotechnologists for their optimum synthesis. Microorganisms, especially molds have been the most prominent microbe for enzymatic synthesis of the prebiotic oligosaccharides. Since 1980s teeming research work was focused towards isolation of potent microbes for oligosaccharide synthesis. The oligosaccharide production has been successfully attempted employing diverse approach viz. SmF, SSF, immobilization of the intact microbial cells or derived enzymes. The successful attempts have been made to improve the strain through mutations.

These laboratory processes have although recorded successful production of oligosaccharides but scaling up introduces exuberant increase in the cost of production of oligosaccharides. The bio process improvement should be inculcated using cheaper agro-industrial wastes as substrates for oligosaccharide production. To decrease the cost of production following issues have to be addressed: (i) a potent and stable microbial enzyme source is to be fetched (ii) scrutinizing agro-industrial wastes befitting the oligosaccharide production (iii) cheaper alternatives for purification strategies of synthesized oligosaccharides.

## Future prospects

As stated in the introduction of the review the demand of health promoting food is expected to rise up to US $4.8 billion by 2018. The hike in the demand is indicative of the future directions towards which the food industry is fastly marching. The so called health promoting food or pro and prebiotics under the unanimous label of “Nutraceuticals” will be a focus of attraction for every such layman growing conscious about health in near future. The present scenario of the health market trend is facing certain health issues pertaining to intake of the prebiotics viz. aggravation of intolerance to lactose, increments in allergic responsiveness of sensitive individuals as reported in several human based case studies.

Looking forward with this setback associated with probiotics, prebiotic are coming up as more promising option. Above all the prebiotic effect of oligosaccharides are now extended to their antidiarrheal, antiobesity and presently towards suppression of type 2 diabetes. The future would really depend on the synergistic effect developed by combinational use of prebiotics and probiotics. The incremental benefits of synbiotics would be auxiliary to the nature’s boon.

## Abbreviations

ADMO: algae derived marine oligosaccharides
AOS: arabinooligosaccharides
CD: cyclodextrins
dp: degree of polymerization
FOSHU: food of specified health use
FOS: fructooligosaccharides
GOS: galactooligosaccharides
GIA: Global Industry Analyst
HMO: human milk oligosaccharides
LDGOS: lactulose derived galactooligosaccharides
MOS: maltooligosaccharides
pAOS: pectin-derived acidic oligosaccharides
XOS: xylooligosaccharides

## References

[CR1] Affertsholt-Allen T. Market developments and industry challenges for lactose and lactose derivatives. IDF Symposium “Lactose and its Derivatives.” Moscow 2007. 2009. http://lactose.ru/present/1Tage_Affertsholt-Allen.pdf.

[CR2] Algieri F, Nogales AR, Mesa NG, Vezza T, Mesa JG, Utrilla MP, Montilla A, Cobas AC, Olano A, Corzo N, Hernández EG, Zarzuelo A, Cabezas MER, Galvez J (2014). Intestinal anti-inflammatory effects of oligosaccharides derived from lactulose in the trinitrobenzenesulfonic Acid model of rat colitis. J Agric Food Chem.

[CR3] Al-Tamimi MAHM, Palframan RJ, Cooper JM (2006). In vitro fermentation of sugar beet arabinan and arabinooligosaccharides by the human gut microflora. J Appl Microbiol.

[CR4] Antosova M, Polakovic M (2001). Fructosyltrasferase : the enzyme catalyzing production of fructooligosaccharides. Chem Pap.

[CR5] Astray G, Gonzalez BC, Mejuto JC, Rial OR, Simal GJ (2009). A review on the use of cyclodextrins in foods. Food Hydrocoll.

[CR6] Backhed F, Ley RE, Sonnenburg JL, Peterson DA, Gordon JI (2005). Host-bacterial mutualism in the human intestine. Science.

[CR7] Barra L, Chandrasekaran R, Corato F, Brunet C (2014). The challenge of ecophysiological biodiversity for biotechnological applications of marine microalgae. Mar Drugs.

[CR8] Beldman G, Schols HA, Pitson SM, Searle-van Leeuwen MJF, Voragen AGJ (1997). Arabinans and arabinan degrading enzymes. Adv Macromol Carbohydr Res.

[CR9] Belorkar SA, Gupta AK, Rai V (2013). Isolation of potential microbial producers of fructosyltransferase from baggasse and selected soil sites of Chhattisgarh, India. Asian J Microbiol Biotechnol Environ Exp Sci.

[CR10] Bernard H, Desseyn JL, Bartke N, Kleinjans L, Stahl B, Belzer C, Knol J, Gottrand F, Husson MO (2015). Dietary pectin-derived acidic oligosaccharides improve the pulmonary bacterial clearance of Pseudomonas aeruginosa lung infection in mice by modulating intestinal microbiota and immunity. J Infect Dis..

[CR11] Bornet RJF, Meflah K, Menanteau J (2002). Enhancement of gut immune functions by short-chain fructooligosaccharides and reduction of colon cancer risk. Biosci Microflora.

[CR12] Casci T, Rastall RA, Gibson GR, Shetty K, Paliyath G, Pometto A, Levin RE (2006). Human gut microflora in health and disease: focus on prebiotics. Food biotechnology.

[CR13] Chen HL, Lu YH, Lin J, Ko LY (2000). Effects of fructooligosaccharide on bowel function and indicators of nutritional status in constipated elderly men. Nutr Res.

[CR14] Chonan O, Takahashi R, Watanuki M (2001). Role of activity of gastrointestinal microflora in absorption of calcium and magnesium in rats fed β1- >4 linked galactooligosaccharides. Biosci Biotechnol Biochem.

[CR15] Chung Y, Hsu C, Ko C (2007). Dietary intake of xylooligosaccharides improves the intestinal microbiota, fecal moisture, and pH Value in the elderly. Nutr Res.

[CR16] Cibe. Environmental report: beet growing and sugar production in Europe. Confederation of European beet grower. Paris, France; 2003.

[CR17] Cote GL (2009). Acceptor products of alternant sucrase with gentiobiose. Production of novel oligosaccharides for food and feed and elimination of bitterness. Carbohydr Res.

[CR18] Courtois J (2009). Oligosaccharides from land plants and algae: production and applications in therapeutics and biotechnology. Curr Opin Microbiol.

[CR19] Crittenden RG, Playne MJ (1996). Production, properties and applications of food-grade oligosaccharides. Trends Food Sci Technol.

[CR20] Dey PM (1983). Further characterization of β-l-arabinosidase from *Cajanus indicus*. Biochim Biophys Acta.

[CR21] Deguchi Y, Matsumoto K, Ito T, Watanuki M (1997). Effects of β1-4 galacto-oligosaccharides administration on defecation of healthy volunteers with constipation tendency. Jpn J Nutr..

[CR22] Dimitroglou A, Merrifield DL, Spring P, Sweetman J, Moate R, Davies SJ (2010). Effects of mannan oligosaccharide (MOS) supplementation on growth performance feed utilization, intestinal histology and gut micro biota of gilthead sea bream (Sparusaurata). Aquaculture.

[CR23] Eseceli H, Demir E, Degirmencioglu N, Bilgic M (2010). The effects of Bio-Mosmannan oligosaccharide and antibiotic growth promoter performance of broilers. J Anim Vet Adv.

[CR24] Fernandez RC, Maresma BG, Juarez A, Martinez J (2003). Production of fructooligosaccharides by β-fructofuranosidase from *Aspergillus* sp. 27 H. J Chem Technol Biotechnol.

[CR25] Flamm G, Glinsmann W, Kritchevsky D, Prosky L, Roberfroid M (2001). Inulin and oligofructose as dietary fiber: a review of the evidence. Crit Rev Food Sci Nutr.

[CR26] Flint HJ, Duncan SH, Scott KP, Louis P (2007). Interactions and competition within the microbial community of the human colon: links between diet and health. Environ Microbiol.

[CR27] Fujimoto Y, Hattori T, Uno S, Murata T, Usui T (2009). Enzymatic synthesis of gentiooligosaccharides by transglycosylation with β-glycosidases from *Penicillium multicolour*. Carbohydr Res.

[CR28] Ganaje MA, Lateef A, Gupta US (2014). Enzymatic trends of fructooligosaccharides production by microorganisms. Appl Biotechnol.

[CR29] Gómez B, Miguez B, Veiga A (2015). Production, purification and in vitro evaluation of the prebiotic potential of arabinoxylooligosaccharides from brewer’s spent grain. J Agric Food Chem.

[CR30] González A, Castro J, Vera J, Moenne A (2012). Seaweed oligosaccharides stimulate plant growth by enhancing carbon and nitrogen assimilation, basal metabolism and cell division. J Plant Growth Regul.

[CR31] Goulas A, Tzortzis G, Gibson GR (2007). Development of a process for the production and purification of α- and β-galactooligosaccharides from *Bifidobacterium bifidum* NCIMB 41171. Int Dairy J.

[CR32] Gour D (2013). Value added dairy products: catalyst for good health. Int Index Refereed J.

[CR33] Guerrero C, Vera C, Illanes A (2013). Optimisation of synthesis of oligo-saccharides derived from lactulose (fructosyl-galacto-oligosaccharides) with β-galactosidases of different origin. Food Chem.

[CR34] Guerrero C, Vera C, Conejeros R, Illanes A (2015). Transgalactosylation and hydrolytic activities of commercial preparations of Î²-galactosidase for the synthesis of prebiotic carbohydrates. Enzym Microb Technol..

[CR35] Gullón P, González-Muñoz MJ, Parajó JC (2011). Manufacture and prebiotic potential of oligosaccharides derived from industrial solid wastes. Bioresou Technol.

[CR36] Hamed I, Özogul F, Özogul Y, Regenstein JM (2015). Marine bioactive compounds and their health benefits:are view. Compr Rev Food Sci Food Saf..

[CR37] Hernandez MLV, Aguirre VMB, Patino ABP, Juarez MC, Moctezuma MPC, Alarcon JJV (2009). Microbial fructosyltransferase and the role of fructans. J Appl Microbiol.

[CR38] Hotchkiss AT, Nunez A, Rastall RA. Growth promotion of beneficial bacteria in gut of human comprises administering composition comprising arabino oligosaccharide as prebiotic US patent 316766–A1; 2010.

[CR39] Hu B, Gong Q, Wang Y, Ma Y, Li J, Yu W (2006). Prebiotic effects of neoagaro-oligosaccharides prepared by enzymatic hydrolysis of agarose. Anaerobe..

[CR40] Iji PA, Kadam MM, Dominguez H (2013). Prebiotic properties of algae and algae- supplemented products A2-Domínguez, Herminia. In functional ingredients from algae for foods and nutraceuticals.

[CR41] Jian W, Sun Y, Huang H, Yang Y, Peng S, Xiong B, Pan T, Xu Z, He M, Pang J (2013). Study on preparation and separation of Konjac oligosaccharides. Carbohydr Polym..

[CR42] Johnson CR, Thavarajah D, Combs GF, Thavarajah R (2013). Lentil (*Lens culinaris* L.): a prebiotic-rich whole food legume. Food Res Int.

[CR43] Kaji A, Saheki T (1975). Endo-arabinase from *Bacillus subtilis* F-11. Biochim Biophys Acta.

[CR44] Kaji A, Shimokawa K (1984). New exo-type arabinase from *Erwinia* carotovora IAM. Agric Biol Chem.

[CR45] Kaji A, Tagawa K (1970). Purification, crystalization and amino acid composition of α-l-arabinofuranosidase from *Aspergillus niger*. Biochim Biophys Acta.

[CR46] Katapodis P, Chistakopoulos P (2008). Enzymatic production of feruloylxylo-oligosaccharides from corn cobs by a family 10 xylanase from *Thermoascusaurantiacus*. LWT Food Sci Technol.

[CR47] Katapodis P, Kalogeris E, Kekos D, Macris BJ (2004). Biosynthesis of fructo-oligosaccharides by *Sporotrichum* *thermophile* during submerged batch cultivation in high sucrose media. Appl Microbiol Biotechnol.

[CR48] Ketabi LA, Dieleman MGG (2011). Influence of isomalto-oligosaccharides on intestinal microbiota in rats. J Appl Microbiol.

[CR49] Kim SK (2013). Marine nutraceuticals: prospects and perspectives.

[CR51] Klaenhammer TR, Barrangou R, Buck BL, Peril MAA, Altermann E (2005). Genomic features of lactic acid bacteria effecting bioprocessing and health. FEMS Microbiol Rev.

[CR52] Kok N, Roberfroid M, Robert A, Delzenne N (1996). Involvement of lipogenesis in lower VLDL secretion induced by oligofructose in rats. Br J Nutr.

[CR53] Komae K, Kaji A, Sato M (1982). An α-l-arabinofuranosidase from *Streptomyces purpurascens* IFO 3389. Agric Biol Chem.

[CR54] Kormelink FJM, Searle-van Leeuwen MJF, Wood TM, Voragen AGJ (1991). Purification and characterization of an (1,4)-β-d-arabinoxylan arabinofuranohydrolase from *Aspergillus awamori*. Appl Microbiol Biotechnol.

[CR55] Lecerf JM, Depeint F, Clerc E (2012). Xylo-oligosaccharide (XOS) in combination with inulin modulates both the intestinal environment and immune status in healthy subjects, while XOS alone only shows prebiotic properties. Br J Nutr.

[CR56] Liong MT, Shah NP (2005). Optimization of cholesterol removal, growth and fermentation patterns of *Lactobacillus acidophilus* ATCC4962 in the presence of mannitol, fructo-oligosaccharide and inulin: a response surface methodology approach. J Appl Microbiol.

[CR57] Liu HT, He JL, Li WM, Yang Z, Wang YX, Bai XF, Yu C, Du YG (2010). Chitosan oligosaccharides protect human umbilical vein endothelial cells from hydrogen peroxide induced apoptosis. Carbohydr Polym.

[CR58] Lordan S, Ross RP, Stanton C (2011). Marine bioactives as functional food ingredients: potential to reduce the incidence of chronic diseases. Mar Drugs.

[CR59] Losada MA, Olleros T (2002). Towards healthier diet for the colon: the influence of fructooligosaccahides and *Lactobacilli* on intestinal health. Nutri Res.

[CR60] Lupp C, Finlay BB (2005). Intestinal microbiota. Curr Biol.

[CR61] Macfarlane GT, Steed H, Macfarlane S (2008). Bacterial metabolism and health-related effects of galacto-oligosaccharides and other prebiotics. J Appl Microbiol.

[CR62] Madhukumar MS, Muralikrishna G (2010). Structural characterization and determination of prebiotic activity of purified xylooligosaccharides obtained from Bengal gram husk (*Cicer arietinum* L.) and wheat bran (*Triticum aestivum*). Food Chem.

[CR63] Maischberger T, Nguyen TH, Sukyai P, Kittl R, Riva S, Ludwig R, Haltrich D (2008). Production of lactose-free galacto-oligosaccharide mixtures: comparison of two cellobiose dehydrogenases for the selective oxidation of lactose to lactobionic acid. Carbohydr Res.

[CR64] Matsubara Y, Iwasaki KI, Nakajima M, Nabetani H, Nakaq SI (1996). Recovery of oligosaccharides from steamed soybean waste water in Tofu processing by reverse osmosis and nanofiltration membrane. Biosci Biotechnol Biochem.

[CR65] McKie VA, Black GW, Millward-Sadler SJ, Hazlewood GP, Laurie JI, Gilbert HJ (1997). Arabinanase A from *Pseudomonas fluorescens* subsp. *cellulosa* exhibits both an endo- and an exo-mode of action. Biochem J.

[CR66] Menrad K (2003). Market and marketing of functional foods in Europe. J Food Eng.

[CR67] Milinki E, Molnár S, Kiss A, Virág D, Pénzes-Kónya E (2011). Study of microelement accumulating characteristics of microalgae. Acta Bot Hung..

[CR68] Moore N, Chao C, Yang LP, Storm H, Oliva HM, Saavedra JM (2003). Effects of fructo-oligosaccharide-supplemented infant cereal: a double-blind, randomized trial. Br J Nutr.

[CR69] Moure A, Gullon P, Dominguez H, Parajo JC (2006). Advances in the manufacture, purification and applications of xylo-oligosaccharides as food additives and nutraceuticals. Process Biochem.

[CR70] Mussatto SI, Aguilar CN, Rodrigues LR, Teixeira JA (2009). Fructooligosaccharides and β-fructofuranosidase production by *Aspergillus japonicus* immobilized on lignocellulosic materials. J Mol Catal B Enzym.

[CR71] Nguyen TH, Splechtna B, Krasteva S, Kneifel W, Kulbe KD, Divne C, Haltrich D (2009). Characterization and molecular cloning of a heterodimeric beta-galactosidase from the probiotic strain *Lactobacillus acidophilus* R22. FEMS Microbiol Lett.

[CR72] O’sullivan L, Murphy B, Mcloughlin P, Duggan P, Lawlor PG, Hughes H (2010). Prebiotics from marine macroalgae for human and animal health applications. Mar Drugs.

[CR73] Palframan R, Gibson GR, Rastall RA (2003). Effect of pH and dose on the growth of gut bacteria on prebiotic carbohydrates in vitro. Anaerobe.

[CR74] Palframan RJ, Gibson GR, Rastall RA (2002). Effect of pH and dose on the growth of gut bacteria on prebiotic carbohydrates in vitro. Anaerobe.

[CR75] Patel S, Goyal A (2010). Functional oligosaccharides: production, properties and applications. World J Microbiol Biotechnol.

[CR76] Patel S, Goyal A (2011). Functional oligosaccharides: production, properties and applications. World J Microbial Biotechnol.

[CR77] Perrin S, Warchol M, Grill JP, Schneider F (2001). Fermentations of fructooligosaccharides and their components by *Bifidobacteriuminfantis* ATCC 15697 on batch culture in semi-synthetic medium. J Appl Microbiol.

[CR78] Placier G, Watzlawick H, Rabiller C, Mattes R (2009). Evolved beta-galactosidases from geobacillus stearothermophilus with improved transgalactosylation yield for galacto-oligosaccharide production. Appl Environ Microbiol.

[CR79] Playne MJ, Crittenden RG, McSweeney PLH, Fox PF (2009). Galacto-oligosaccharides and other products derived from lactose. Lactose, water, salts and minor constituents.

[CR80] Prapulla SG, Subhaprada V, Karanth NG (2000). Microbial production of oligosaccharides: a review. Adv Appl Microbiol.

[CR81] Quigley EMM (2010). Prebiotics and probiotics; modifying and mining the microbiota. Pharmacol Res.

[CR82] Rastall RA, Hotchkiss AT, Gillian E, Cote` GL (2003). Potential for the development of prebiotic oligosaccharides from biomass. Oligosaccharides in food and agriculture.

[CR83] Roberfroid M (2000). Prebiotics and probiotics: are they functional foods?. Am J Clin Nutr.

[CR84] Roberfroid M, Slavin J (2000). Non-digestible oligosaccharides. Crit Rev Food Sci Nutr.

[CR85] Rombouts FM, Voragen AGJ, Searle-van Leeuwen MF, Geraerds CCJM, Schols HA, Pilnik W (1988). The arabinanases of *Aspergillus niger*—purification and characterisation of two α-l-arabinofuranosidases and an *endo*-1,5-α-l-arabinanase. Carbohydr Polym.

[CR86] Rudi K (2010). Dynamic host–bacteria interactions during an acidotic state induction. Environ Microbiol Rep.

[CR87] Rycroft CE, Jones MR, Gibson GR (2001). A comparative in vitro evaluation of the fermentation properties of prebiotic oligosaccharides. J Appl Microbiol.

[CR88] Sako T, Matsumoto K, Tanaka R (1999). Recent progress on research and applications of nondigestible galacto-oligosaccharides. Int Dairy J.

[CR89] Saminathan M, Sieo CC, Kalavathy R, Abdullah N, Ho YW (2011). Effect of prebiotic oligosaccharides on growth of *Lactobacillus* strains used as a probiotic for chickens. Afr J Microbiol Res.

[CR90] Sanchez O, Guio F, Garcia D, Silva E, Caicedo L (2008). Fructooligosaccharides production by *Aspergillus* sp. N74 in a mechanically agitated airlift reactor. Food Bioprod Process.

[CR91] Sangeetha PT, Ramesh MN, Prapulla SG (2005). Recent trends in the microbial production, analysis and application of fructooligosaccharides. Trends Food Sci Technol.

[CR92] Sanz ML, Gibson GR, Rastall RA (2005). Influence of disaccharide structure on prebiotic selectivity in vitro. J Agric Food Chem.

[CR93] Sheu WHH, Lee IT, Chen W (2008). Effects of xylooligosaccharides in type 2 diabetes mellitus. J Nutr Sci Vitaminol.

[CR94] Sanz SL, Montilia A, Moreno FJ, Villamiel M (2015). Stability of oligosaccharides derived from lactulose during the processing of milk and apple juice. Food Chem.

[CR95] Shuhaimi M, Kabier BM, Yazid AM, Somchit MN (2009). Synbiotics growth optimization of *Bifidobacteriumpseudocatenulatum* G4 with prebiotics using a statistical methodology. J Appl Microbiol.

[CR96] Slevin MM, Allsopp PJ, Magee PJ, Bonham MP, Naughton VR, Strain JJ, Duffy ME, Wallace JM, Mac Sorley EM (2014). Supplementation with calcium and short-chain fructooligosaccharides affects markers of bone turnover but not bone mineral density in postmenopausal women. J Nutr.

[CR97] Spinner J. Prebiotics market to hit $ 4.8 billion by 2018. Newsletter–food production daily.com. 2013. http://www.foodproductiondaily.com/Financial/Prebiotics-market-to-hit-4.8-billion.

[CR98] Splechtna B, Nguyen TH, Steinbock M, Kulbe KD, Lorenz W, Haltrich D (2006). Production of prebiotic galacto-oligosaccharides from lactose using beta-galactosidases from *Lactobacillus reuteri*. J Agric Food Chem.

[CR001] Splechtna B, Nguyen TH, Haltrich D (2007). Comparison between discontinuous and continuous lactose conversion processes for the production of prebiotic galacto-oligosaccharides using beta-galactosidase from *Lactobacillus reuteri*. J Agric Food Chem.

[CR99] Stengel DB, Connan S, Popper ZA (2011). Algal chemodiversity and bioactivity: sources of natural variability and implications for commercial application. Biotechnol Adv.

[CR100] Sulek K, Vigsnaes LK, Schmidt LR (2014). A combined metabolomic and phylogenetic study reveals putatively prebiotic effects of high molecular weight arabino-oligosaccharides when assessed by in vitro fermentation in bacterial communities derived from humans. Anaerobe.

[CR101] Topping DL, Clifton PM (2001). Short-chain fatty acids and human colonic function: roles of resistant starch and nonstarch polysaccharides. Physiol Rev.

[CR102] Van Laere KMJ, Beldman G, Voragen AGJ (1997). A new arabinofuranohydrolase from *Bifidobacterium adolescentis* able to remove arabinosyl residues from double-substituted xylose units in arabinoxylan. Appl Microbiol Biotechnol.

[CR103] Vander MR, Avonts L, De VL (2004). Short fractions of oligofructose are preferentially metabolized by *Bifidobacteriumanimalis* DN-173 010. Appl Environ Microbiol.

[CR104] Vigsnæs LK, Holck J, Meyer AS (2011). In vitro fermentation of sugar beet arabino-oligosaccharides by fecal microbiota obtained from patients with ulcerative colitis to selectively stimulate the growth of *Bifidobacterium* spp. and *Lactobacillus* spp. Appl Env Microbiol..

[CR105] Vogel M (1991). Alternative utilisation of sugar beet pulp. Zuckerindustrie.

[CR106] Voragen AGJ, Rombouts FM, Searle-van Leeuwen MF, Schols HA, Pilnik W (1987). The degradation of arabinans by endo-arabinanase and arabinofuranosidases purified from *Aspergillus niger*. Food Hydrocoll.

[CR107] Wallenfels K, Malhotra OP, Boyer PD (1960). Beta-galactosidase. The enzymes.

[CR108] Wang P, Jiang X, Jiang Y, Hu X, Mou H, Li M (2007). In vitro antioxidative activities of three marine oligosaccharides. Nat Prod Res.

[CR109] Weinstein L, Alber sheim P (1979). Structure of plant cell walls. IX. Purification and partial characterization of a wall-degrading endo-arabanase and an arabinosidase from *Bacillus subtilis*. Plant Physiol.

[CR110] Westphal Y, Kuhnel S, Waard P, Schols SWA, Schols HA, Voragen AGJ, Gruppen H (2010). Branched arabino-oligosaccharides isolated from sugar beet arabinan. Carbohydr Res.

[CR111] Wu SC, Wen TN, Pan CL (2005). Algal-oligosaccharide-lysates prepared by two bacterial agarases stepwise hydrolyzed and their anti-oxidative properties. Fish Science.

[CR112] Yoo HD, Kim D, Park SH (2012). Plant cell wall polysaccharides as potential resources for the development of novel prebiotics. Biomol Ther (Seoul).

